# Molecular Evidences of a Hidden Complex Scenario in *Leporinus* cf. *friderici*

**DOI:** 10.3389/fgene.2018.00047

**Published:** 2018-02-15

**Authors:** Rosane Silva-Santos, Jorge L. Ramirez, Pedro M. Galetti, Patrícia D. Freitas

**Affiliations:** Laboratório de Biodiversidade Molecular e Conservação, Departamento de Genética e Evolução, Universidade Federal de São Carlos, São Paulo, Brazil

**Keywords:** neotropical fish, MOTUs, fish phylogeny, taxonomic uncertainties, cryptic species

## Abstract

The megadiversity of the neotropical ichthyofauna has been associated to recent diversification processes, reflecting in subtle or lacking morphological differentiation between species, challenging the classical taxonomic identification. *Leporinus friderici* occurs in several river basins of South America, and its nominal taxonomic validity has been questioned. Its wide distribution within the Brazilian Shield suggests that this species could be genetically structured among the hydrographic basins, despite a sharp morphological similarity. In this study, we performed phylogenetic analyses, based on three nuclear (recombination activating gene 1, RAG1, recombination activating gene 2, RAG2, and myosin heavy chain 6 cardiac muscle alpha gene, Myh6) and two mitochondrial (COI and Cytochrome b, Cytb) markers, in specimens morphologically similar to *L. friderici* and related species from different hydrographic basins in South America. Our phylogenetic tree identified four well-supported clades, which point out to the existence of taxonomic inconsistencies within this fish group. A clade named *L*. cf. *friderici sensu stricto* included eight Molecular Operational Taxonomic Units recently diversified in the Brazilian Shield basins. These results were also confirmed by a single-gene species delimitation analysis. It is suggested that this clade includes a species complex, characterizing taxonomic uncertainties. Another clade recovered only *L. friderici* from the Suriname rivers, validating this nominal species in its type locality. A third no-named clade, characterized by deeper species divergence, recovered five different nominal species interleaved with other undescribed forms previously also recognized as *L.* cf. *friderici*, indicating taxonomic errors. The fourth clade only included *L. taeniatus*. Our results showed a complex scenario involving the morphotype *L.* cf. *friderici* and allowed us to address aspects related to evolutionary diversification of this fish group and historical processes involved with, highlighting the importance of revealing hidden biodiversity for the taxonomy and conservationist action plans of these fish.

## Introduction

South America freshwater fish represent one-third of the world continental ichthyofauna (Reis et al., 2016). However, this huge biodiversity is relatively recent, mostly due to extensive speciation events during the last 10 Ma (Hubert et al., 2007; Albert and Reis, 2011). Several taxa diverged <1 Ma during the late Pleistocene because of the Quaternary activity that led to topographic changes such as recent drainage rearrangements (Ribeiro, 2006). These events probably reflected in subtle or lacking morphological differentiation between the emerged species, challenging the classical taxonomic identification.

Molecular analyses have been largely used to aid species identification and delimitation within neotropical fish (e.g., Carvalho et al., 2011; Pereira et al., 2011, 2013; Ramirez and Galetti, 2015; Machado et al., 2016), contributing in revealing hidden biodiversity (Pires et al., 2017; Ramirez et al., 2017a). Both DNA barcoding and phylogeny studies have provided important contributions for better understand the phylogenetic relationships within taxa, and historical and evolutionary processes involved in the species diversification (e.g., Hebert et al., 2003; Carvalho-Costa et al., 2011; Ramirez et al., 2017b). In this way, mitochondrial and nuclear DNA data can be used to define discrete genetic lineages, detecting reciprocal monophyly, and characterizing a Molecular Operational Taxonomic Unit (MOTU, cluster of orthologous sequences generated by an explicit algorithm), representing a monophyletic lineage that could or not correspond to a taxa (Blaxter et al., 2005; Jones et al., 2011).

*Leporinus*, from the Anostomidae family, is considered one of the richest genus within Characiformes, a predominant freshwater fish order in South America (Garavello and Britski, 2003). Recent morphological and molecular studies have shown *Leporinus* as a non-monophyletic genus (Sidlauskas and Vari, 2008; Ramirez et al., 2016), highlighting the need of a deep taxonomic revision on this group. Freshly, an integrated morphological, chromosomal, and molecular approach described the new genus *Megaleporinus*, gathering the largest body sized *Leporinus* species in a monophyletic clade (Ramirez et al., 2017b).

Among the remaining *Leporinus* species, *Leporinus friderici* Block (1794) has a wide geographic distribution occurring in most rivers of South America (Garavello et al., 1992). This large distribution has been investigated by several authors, who have found morphological (Géry et al., 1987; Renno et al., 1990, 1991; Garavello et al., 1992; Sidlauskas and Vari, 2012) and genetic (Renno et al., 1990, 1991) variations among populations, suggesting that this *L. friderici* morphotype may contain a species complex (Sidlauskas and Vari, 2012). While *L. friderici* from Suriname and French Guiana rivers has been recognized as the type species (Sidlauskas and Vari, 2012), the provisional nomenclature *Leporinus* cf. *friderici* has been used to refer to the remaining individuals of this morphotype. However, because of its wide distribution within the Brazilian Shield, one could expect some levels of genetic differentiation within the *L.* cf. *friderici* among hydrographic basins. Morphological variations in the eyes, body length, and color patterns have been already reported between *L.* cf. *friderici* from the Amazon and Paraná-Paraguay basins (Géry et al., 1987; Garavello et al., 1992).

In this study we tested the hypothesis that *L.* cf. *friderici* consists of a monophyletic group formed by different MOTUs currently separated in distinct basins within the Brazilian Shield. We performed a phylogenetic analysis using mitochondrial and nuclear markers to confirm *L.* cf. *friderici* as a monophyletic group, and a single-gene species delimitation analysis to characterize MOTUs. Finally, we infer on the evolutionary historical processes possibly responsible for the diversification within this group.

## Materials and Methods

### Ethics Statements

This research was conducted within the protocols approved by the Ethics Committee on Animal Experimentation (CEUA, Federal University of São Carlos, São Carlos, São Paulo, Brazil) and SISBIO-ICMBio (Authorization System and Biodiversity Information, Chico Mendes Institute for Biodiversity Conservation, Ministry of Environment, Brazil). The biological samples were obtained under CMBIO/MMA No. 32215 and CEUA No. 3893250615 permits following all legal requirements. Samples from Colombia and Peru were obtained by Jose Ariel Rodriguez and Hernan Ortega, respectively, who provided DNA aliquots for this study.

### Biological Specimens and Data Sampling

**FIGURE 1:**
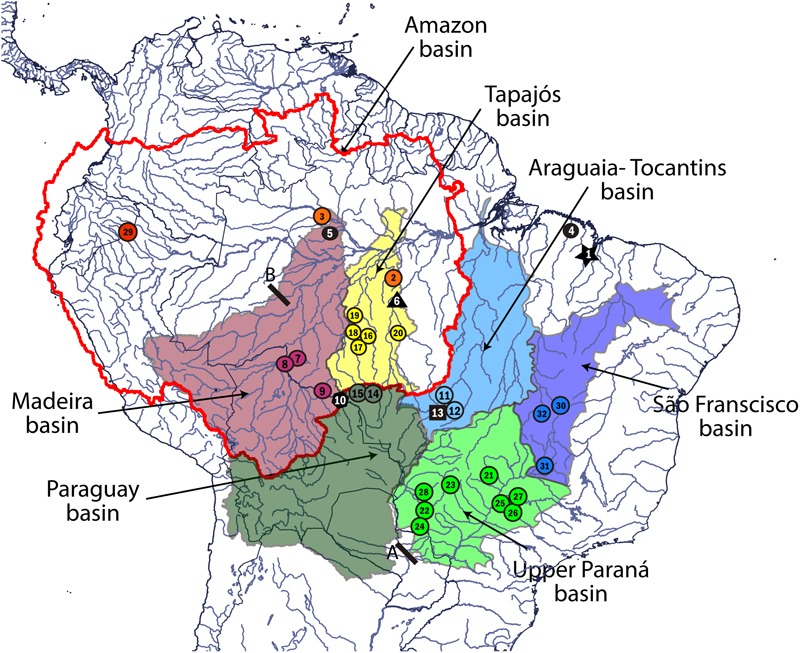
Collection sites and hydrographic basin of occurrence of *Leporinus* cf. *friderici*. Colored circles represent the MOTUs found in this study: *L. agassizii* (red), *L.* cf. *friderici* Amazon 1 (orange), *L.* cf. *friderici* Madeira 1 (purple), *L.* cf. *friderici* Paraná (light green), *L.* cf. *friderici* Paraguay (dark green), *L. piau* São Francisco (dark blue), *L.* cf. *friderici* Upper Tapajós (yellow), and *L.* cf. *friderici* Tocantins 1 (light blue). Black dot represents individuals collected as *L.* cf. *friderici* Amazon 2; black star represents *L.* cf. *friderici* Mearim; black square represents *L.* cf. *friderici* Tocantins 2; black polygon represents *L.* cf. *friderici* Madeira 2; black triangle represents *L.* cf. *friderici* Xingú. A, Prior Sete Quedas waterfalls region and Itaipú dam. B, Teotônio falls in the Madeira River. Map created by Q-GIS (http://www.qgis.org/). The collection site numbers are according to **Supplementary Table S1**.

Samples of fin, muscle, or liver tissues were collected of 53 *L.* cf. *friderici* specimens from eight hydrographic basins from the Brazilian Shield. **Figure 1** illustrates the studied river basins. Samples of *Leporinus agassizii* Steindachner (1876), *L. boehlkei* Garavello (1988), and *L. piau* Fowler (1941), from Jaguaribe and São Francisco rivers and *L*. cf. *parae* Eigenmann and Ogle (1907), were also obtained. In addition, DNA sequences of *Leporinus desmotes* Fowler (1941), *Leporinus fasciatus* Block (1794), *Leporinus lacustris* Campos (1945), *Leporinus octomaculatus* Britski and Garavello (1993), *Leporinus taeniatus* Lütken (1875), *Leporinus venerei* Britski and Birindelli (2008), and *Hypomasticus pachycheilus* Britski (1976), were downloaded from GenBank (Ramirez et al., 2016). All these species were included in our study because they have been considered as closely related to *L. friderici* (Garavello, 1988; Britski and Birindelli, 2008; Ramirez et al., 2016). Sequences of *L. friderici* from Suriname, that is considered the type locality, were obtained from GenBank (Melo et al., 2016) as well. Information related to the specimens, vouchers, IDs, and site localities are recorded in the Supplementary Material (**Supplementary Tables S1, S2**).

### DNA Extraction, Gene Amplification, and Sequencing

Total DNA was extracted using the conventional phenol–chloroform/proteinase K protocol (Sambrook et al., 1989). For the phylogenetic analysis at least one individual per each major sampled hydrographic basin was used. Cytochrome b (Cytb) and cytochrome oxidase subunit 1 (COI) mitochondrial regions were amplified according to Ramirez and Galetti (2015). The nuclear recombination activating gene 1 (RAG1), recombination activating gene 2 (RAG2), and myosin heavy chain 6 cardiac muscle alpha gene (Myh6) were amplified following Oliveira et al. (2011). PCR products obtained for both DNA strands were sequenced on an ABI 373xl sequencer (Applied Biosystems, Little Chalfont, United Kingdom).

### Data Analysis

The obtained sequences were manually edited and aligned using Bioedit (Hall, 1999) and Clustal W (Thompson et al., 1994), respectively. All sequences were checked for indels and stop codons. The haplotypes of the nuclear genes were combined into a consensus sequence by coding polymorphic sites with the IUPAC ambiguity codes (IUPAC, 1974).

The phylogenetic analyses were conducted for concatenated sequences of all mitochondrial and nuclear genes using maximum parsimony (MP), implemented in PAUP^∗^4.0 (Swofford, 2003), with 1000 bootstrap replicates. We also performed maximum-likelihood (ML) analyses, using RAxML in XSEDE (Stamatakis, 2006; Stamatakis et al., 2008), through the web server CIPRES Science Gateway (Miller et al., 2010), and a partitioned model determined by PartitionFinder (Lanfear et al., 2012), under a GTR+G model, and 1000 bootstrap replicates.

A multilocus Bayesian species tree (BST) was estimated by ^∗^BEAST (Star-BEAST) (Heled and Drummond, 2010) using 150 million generations, sampled every 5000, and a burn-in of 300. A nucleotide substitution model selected was based on the Bayesian criterion, using JModeltest 2 (Darriba et al., 2012). The models chosen were HKY+I+G, HKY+G, K80+I, K80+I, and K80+I for COI, Cytb, Myh6, RAG1, and RAG2, respectively. The two mitochondrial gene tree topologies were linked and set to have an effective population size of one-quarter from that of nuclear genes. A lognormal relaxed clock was used for all partitions. Bayesian trees using all sequences were established for each gene, separately. These trees were estimated with 10 million of generations, sampling every 5000, and a burn-in of 10%. Yule speciation model was used and nucleotide substitution models followed the same criteria above cited (**Supplementary Figures S1–S5**). The convergence (sample size >200) and stationarity of the values were checked in TRACER v1.6 (Rambaut et al., 2014).

Two different analyses, using a single-gene species delimitation approach based on COI sequences, were performed to determine the number of MOTUs within the clade widely distributed in the Brazilian Shield (*L.* cf. *friderici sensu stricto*, see below). First, the General Mixed Yule-Coalescent (GMYC) model (Pons et al., 2006) was used to determine the clustering of the COI haplotypes. This analysis was performed with a single threshold that was implemented in the *SPLITS* package using R 3.3.3 statistical software (R Core Team, 2017). For this analysis an ultrametric tree was generated using BEAST 2.3.2 (Bouckaert et al., 2014), with a lognormal relaxed clock, a birth and death model, and a HKY substitution model chosen by jModeltest 2 (Darriba et al., 2012). A total of 50 million MCMC generations and a burn-in of 10% were used. In second, a Bayesian analysis of genetic structure was implemented using BAPS software (Corander et al., 2008). The maximum number of genetically diverged groups (*K*) was firstly set up for 10 replicates, 10 times. The obtained groups containing samples from different basins were submitted to a second layer of analysis in BAPS using *K* = 1–3, replicated also 10 times. This hierarchical approach to DNA sequence clustering provides a useful way to increase statistical power and detect separated haplogroups that are assigned to conservative clusters (Cheng et al., 2013). The most likely *K* was chosen based on log (likelihood) and posterior probability values. Next, we consider the concordant groups between the two different analyses, and the groups presenting allopatry and reciprocal monophyly as the final number of MOTUs.

The genetic distances between MOTUS were calculated based on K2P model using MEGA 6.06 (Tamura et al., 2013). Finally, a haplotype network was generated using Median Joining (Bandelt et al., 1999) in the POPART software (Leigh and Bryant, 2015).

## Results

We successful obtained a total of 127 sequences for the specimens studied herein [63 for the COI gene (557 bp) and 16 for each remaining amplified marker – Cytb (1005 bp), Myh6 (754 bp), RAG1 (1477 bp), and RAG2 (1023 bp)]. The GenBank accession numbers are shown in the Supplementary Material section (**Supplementary Table S2**).

**FIGURE 2:**
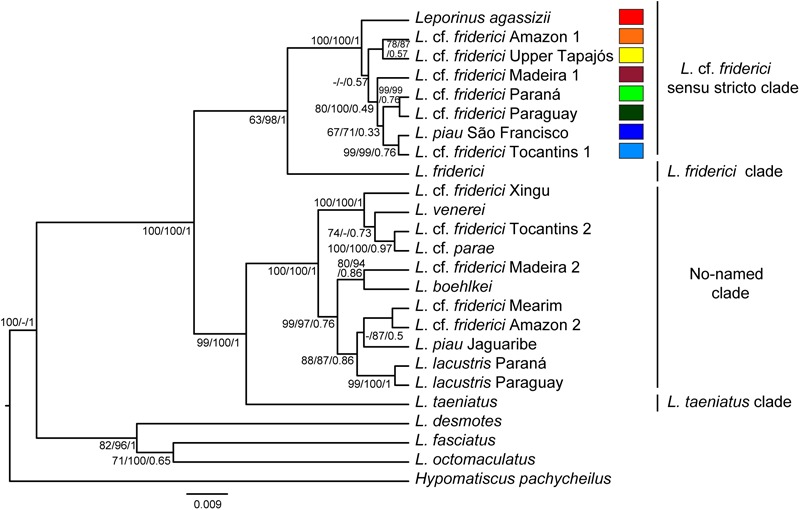
Species tree showing phylogenetic relationships of *Leporinus* cf. *friderici*. Trees were generated using five molecular markers (sequences with approximately 4900 bp). The topology corresponds to Bayesian tree. The numbers on the branches are bootstrap values for maximum parsimony and maximum likelihood, and posterior probability for Bayesian species tree. The scale bar indicates nucleotide substitutions per site.

The phylogenetic trees, generated by MP, ML, and BST analyses (**Figure 2**), strongly supported four clades with maximum support values. A clade recovered *L. friderici* from the Suriname basin alone. A monophyletic clade characterized by a recent diversification within the Brazilian Shield, named herein as *L.* cf. *friderici sensu stricto*, included specimens of *L*. cf. *friderici* from Amazonas (main channel), Madeira, Upper Tapajós, Tocantins, Paraguay, and Paraná basins, *L. agassizii*, and specimens of *L. piau* from São Francisco basin. A no-named clade, showing older diversification, joined specimens of *L*. cf. *friderici*, from Mearim, Tocantins, Turiaçu, Xingu, and Madeira basins, interleaved with *L. boehlkei, L. lacustris, L.* cf. *parae, L. piau* from Jaguaribe basin, and *L. venerei*. Lastly, a fourth clade recovered only *L. taeniatus*.

**FIGURE 3:**
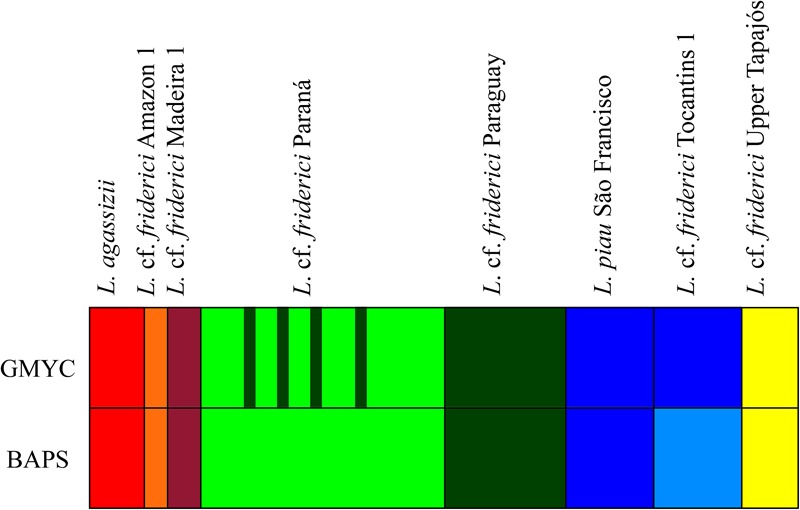
MOTUs identified based on the GMYC and BAPS analyses for *Leporinus* cf. *friderici sensu stricto* clade.

The GMYC analysis, considering 23 parsimony-informative sites and no insertions or deletions within the COI sequences, identified seven MOTUs (CI: 6–7), with a significant likelihood ratio of 10.97 (*P* < 0.005) within *L.* cf. *friderici sensu stricto*. From these, six MOTUs corresponded to *L. agassizii, L.* cf. *friderici* Amazon 1, *L.* cf. *friderici* Madeira 1, *L.* cf. *friderici* Paraná, *L.* cf. *friderici* Paraguay, and *L.* cf. *friderici* Upper Tapajós. The seventh MOTU joined *L. piau* São Francisco and *L.* cf. *friderici* Tocantins 1 (**Figure 3**). The results of the first BAPS layer presented four as the most likely *K* with log (ml) = -554.0008 and 0.93 posterior probability values. In addition, the results of the second BAPS layer were similar to the GMYC MOTUs, but recovered *L. piau* São Francisco and *L.* cf. *friderici* Tocantins as two different MOTUs. The hierarchical analysis of BAPS could separate these populations, since information related to sample locations were given. Differently, GMYC method considers no prior information. Despite this little divergence in detecting MOTUs, both these lineages presenting recent divergence are reciprocally monophyletic and geographically isolated in nature.

The mean genetic distance COI K2P values among the MOTUs ranged from 0.4 to 2.4%. The maximum intra-MOTU distance (0.5%) was observed in *L. agassizii*, while the minimum inter-MOTU distance (0.4%) was between *L.* cf. *friderici* Tocantins and *L. piau* São Francisco (**Supplementary Table S3**).

**FIGURE 4:**
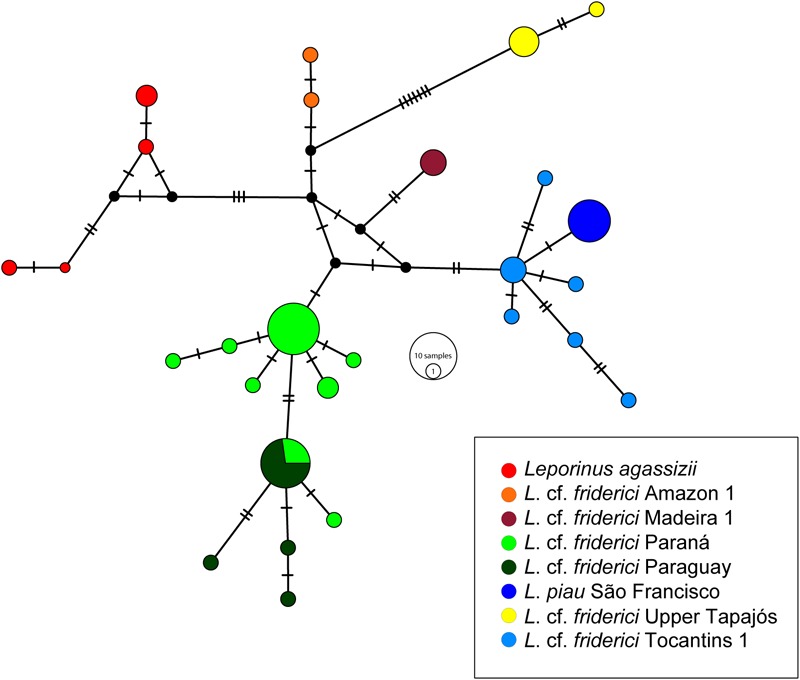
The Median Joining haplotype network of *Leporinus* cf. *friderici sensu stricto* clade using COI gene data. The small dark dots indicate the missing or not sampled haplotypes. The haplotypes are shown in different colors according to each MOTU in legend.

A total of 27 haplotypes was obtained within *L.* cf. *friderici sensu stricto*, in which each MOTU was represented by a haplogroup, except for the MOTUs from Paraná and Paraguay that shared one haplotype. *L. piau* São Francisco and *L.* cf. *friderici* Upper Tapajós were separated by only one mutational step, while the other haplogroups were connected by at least two mutational steps (**Figure 4**).

## Discussion

Our phylogenetic analyses showed that specimens morphologically identified as *L. friderici* constitute a polyphyletic group, widely distributed along the South America (**Figure 2**). The individuals collected as *L. friderici* across the Brazilian Shield basins are not conspecific with *L. friderici* from the type locality, representing a different species. *L.* cf. *friderici sensu stricto* constitutes a monophyletic species complex distributed in Amazon, Madeira, Upper Tapajós, Tocantins, São Francisco, Paraná, and Paraguay river basins. This finding can represent a typical situation of recent diversification forming a strictly related group composed of potential cryptic species, revealing typical taxonomic uncertainties (Ramirez et al., 2017a). On the other hand, in the no-named clade the five nominal species *L. boehlkei, L*. cf. *parae, L. lacustris, L. piau* Jaguaribe, and *L. venerei* were interleaved with individuals that morphologically fit with the description of *L.* cf. *friderici*. In this clade, characterized by an older diversification, the use of the term *L.* cf. *friderici* hides undescribed cryptic species. All species from this clade share discriminative morphological general pattern with *L. friderici* (i.e., one–three spots on the body along the lateral-line, and dental formulae 4/4, except *L. venerei* that has 4/3, unique in Anostomidae), hindering the identification of these cryptic species. Moreover, three (*L. lacustris, L. parae* – *L*. cf. *parae* –, and *L. venerei*) of the five nominal species of this clade have been already considered as very similar in morphology due to their deep body, terminal mouth, anal fin long and dark, and three blotches on the lateral line (Britski and Birindelli, 2008).

Overall, the results obtained for *L.* cf. *friderici sensu stricto* confirm our hypothesis that there are different MOTUs within *L.* cf. *friderici*, currently separated in distinct river basins, but not all provisionally recognized as *L.* cf. *friderici*, that can be joined in a single monophyletic group.

Within the clade *L.* cf. *friderici sensu stricto, L. agassizii* is clearly recognized as a valid species different from *L. friderici*, mainly due to the presence of a longitudinal stripe, extending from dorsal fin to just before the caudal fin (Birindelli et al., 2013). *L. agassizii* was firstly described for the Iça river, Upper Amazon basin (Steindachner, 1876), and posteriorly it was also found in the Tefe lake, Nanay, Negro, and Branco rivers (Birindelli et al., 2013), from the same basin. This species has been described as restricted to the Upper Amazon basin. A parsimony analysis of endemism in the South America reported the Upper Amazon as a separate clade from the Amazon drainages (Hubert and Renno, 2006), suggesting that uplift of the paleoarches has promoted allopatric divergence in the ichthyofauna of this region, which was enhanced by marine incursions.

In our study, all individuals collected in rivers above Madeira river falls were joined in the single MOTU *L*. cf. *friderici* Madeira 1, while individuals downstream the Amazon basin were recovered in the MOTU *L.* cf. *friderici* Amazon 1 (**Figure 3**), with the exception of individuals from Upper Tapajós (see below), indicating these falls as possible barrier that limits the fish species distribution in the region. Previous studies had already reported evidences of structuring along the Amazon basin (Goulding, 1979; de Queiroz et al., 2013). This subdivision was attributed to geomorphological agents that allowed allopatric fragmentation and diversification events (Albert and Reis, 2011). In the Madeira river, the Teotônio fall seems to play a relevant role on the ichthyofauna diversification of the Amazon basin. This fall, besides other rapids, has been keeping apart the rivers from Upper and Lower Madeira, and has been considered a geographic barrier by limiting the fish species distribution in the region (Zanata and Toledo-Piza, 2004; Hubert et al., 2007; Torrente-Vilara et al., 2011).

Barrier effects can also explain the presence of *L.* cf. *friderici* Upper Tapajós joining individuals caught in the Juruena – Teles Pires sub-basin, in the Upper Tapajós (**Supplementary Tables S1, S2**). Waterfalls and rapids along the Tapajós river, and in its tributaries seem acting as barriers to fish dispersal (Britski and Garavello, 2005; Britski and Lima, 2008; Dagosta and de Pinna, 2017). The region above the Juruena – Teles Pires confluence river has been characterized by an endemic ichthyofauna different from other Amazon rivers (Carvalho and Bertaco, 2006; Britski and Lima, 2008), which could account to the separation of *L.* cf. *friderici* Upper Tapajós.

In turn, the Tocantins basin is considered an independent system from the Amazon basin, since its waters flow directly into the Atlantic Ocean (Albert and Reis, 2011). This fact was reflected in our analyses, in which the Tocantins individuals corresponded to a different genetic group (*L.* cf. *friderici* Tocantins 1). The final establishment of modern course of Tocantins (1.8 Ma) separated definitively this basin from Amazon (Rossetti and Valeriano, 2007), and the differentiation of the Tocantins ichthyofauna has been often associated to the rise of Gurupá arch, the Tucurui rapids, or the limited connectivity (Hubert et al., 2007; Hrbek et al., 2014).

The relationship between *L. piau* São Francisco and *L.* cf. *friderici* Tocantins 1 observed here can be accounted for a biogeographic history between the Tocantins and São Francisco basins, and the low genetic divergence (0.4%) between them likely represents a recent diversification. These two hydrographic basins share an extensive watershed, where the Sapão river (São Francisco basin) shares headwaters with the Galheiros river, Tocantins basin (Lima and Caires, 2011). The existence of these common headwaters can allow a fauna exchange between these basins. Geological evidence shows that the western border of Serra Geral from the Goiás plateau has been gradually eroded and could have potentially promoted headwater capture events between the São Francisco and Tocantins rivers (Lima and Caires, 2011). Geodispersal events (i.e., headwater capture) from Amazon river to eastern basins of the Brazilian Shield (as São Francisco river) have been already claimed in studies using molecular approaches (Hubert et al., 2007; Ramirez et al., 2017b).

While the *L. piau* specimens from São Francisco was linked to the *L.* cf. *friderici sensu strito* clade, *L*. *piau* from Jaguaribe was grouped in the no-named clade (**Figure 2**), revealing a clear taxonomic inconsistency. Fowler (1941) claimed the Salgado river (Jaguaribe basin) from Ceará state as type locality of *L. piau*, and included one paratype from Jatobá river (São Francisco basin). Consequently, specimens from São Francisco river have been usually cited as *L. piau* (Garavello and Britski, 2003; Carvalho et al., 2011). Our results pointed that specimens from São Francisco basin indeed constitute a different species from the *L. piau* from the Jaguaribe river, the type basin.

Still within *L.* cf. *frideric*i *sensu stricto*, a well-supported differentiation between specimens from the Upper Paraná and Paraguay basins was also observed, although some individuals from the Upper Paraná showed haplotypes from the Paraguay basin (**Figures 3, 4**). It is possible that both *L.* cf. *friderici* Paraná and *L.* cf. *friderici* Paraguay reached their current distribution through ancient geodispersal events, as headwater captures between Amazon rivers and the Paraná and Paraguay basins. In the modern river basin landscape, the Paraguay basin has a watershed with the Guaporé, Tapajós, and Xingu rivers, while the Upper Paraná shared a watershed at the headwaters of the Tocantins basin (Albert and Reis, 2011). These hydrographic systems have experienced a long history of major capture events and formation of semipermeable barriers (Lundberg et al., 1998) that can support this hypothesis. The Upper Paraná ichthyofauna was separated from the Lower Paraná by the Sete Quedas Falls, a natural geographic barrier which no longer occurs. In the past this barrier isolated the Upper Paraná, where the ichthyofauna has been diverging, as already reported in *Megaleporinus* (Ramirez et al., 2017b) and *Salminus* (Machado et al., 2016). The shared haplotypes between *L.* cf. *friderici* Paraná and *L.* cf. *friderici* Paraguay are probably resulting of the removal of the natural barrier when the Itaipu hydroelectric was built. The resulted dam flooded an extensive area, including the no longer existent Sete Quedas Falls, allowing the connection between both ichthyofauna from Lower and Upper Paraná facilitating contact between mitochondrial lineages since the formation of Sete Quedas Falls (Júlio et al., 2009; Prioli et al., 2012).

## Conclusion

Our study showed that *L.* cf. *friderici* as provisionally used hides at least two major situations. First, *L.* cf. *friderici sensu stricto*, a monophyletic clade joining eight MOTUs, potentially includes a true species complex, characterized by recent diversification across the Brazilian Shield basins. According to our initial expectations, *L.* cf. *friderici sensu stricto* is genetically structured along the Brazilian shield basins, and this structure appears to be related to geomorphological agents, determining the current hydrographic structure. Its taxonomic significance is an open question, requesting complementary studies for resolving this typical situation of taxonomic uncertainties. Second, a no-named clade, characterized by relatively older diversification, in our opinion, hides undescribed cryptic species under *L*. cf. *friderici* denomination, likely due to the morphology similarities that characterize the clades here studied (except *L. taeniatus* clade). However, this new MOTUs show deep phylogenetic divergence and they are interleaved with other nominal valid species (*L. venerei, L. boehlkei, L. lacustris, L. piau*, and *L.* cf. *parae*), supporting them as potential new species.

Overall, our results have important significance for the taxonomy and evolutionary knowledge of this fish group as well as for its conservation. Moreover, this scenario indicates that *L.* cf. *friderici sensu stricto* can constitute an excellent phylogeographic model in studying evolutionary and speciation processes acting in the South America basins. Despite its migratory behavior, *L.* cf. *friderici* cannot be considered as a single genetic stock even within the same basin (i.e., Amazon basin) and needs to be well known for having its whole diversity considered in any conservation effort. For a more complete understanding, the taxonomic status of each MOTU that was revealed herein needs to be evaluated using preferentially morphological and molecular data in an integrative approach.

## Author Contributions

RS-S and JR collected the data, reviewed the literature, and achieved the bioinformatic analyses. All authors contributed to design the research, article writing and discussion, and approved the final version of the manuscript.

## Conflict of Interest Statement

The authors declare that the research was conducted in the absence of any commercial or financial relationships that could be construed as a potential conflict of interest.

## Supplementary Material

FIGURE S1 Bayesian tree for the cytochrome oxidase subunit 1 (COI) gene. Values on nodes represent the posterior probability.

FIGURE S2 Bayesian tree for the cytochrome b (Cytb) gene. Values on nodes represent the posterior probability.

FIGURE S3 Bayesian tree for the myosin heavy chain 6 cardiac muscle alpha (Myh6) gene. Values on nodes represent the posterior probability.

FIGURE S4 Bayesian tree for the recombination activating gene 1 (RAG1) gene. Values on nodes represent the posterior probability.

FIGURE S5 Bayesian tree for the recombination activating gene 2 (RAG2) gene. Values on nodes represent the posterior probability.

TABLE S1 Vouchers and collection sites for all analyzed *Leporinus* cf. *friderici*.

TABLE S2 GenBank accession numbers for all analyzed species.

TABLE S3 Pairwise mean genetic distances values inter and intra-MOTU (in bold) *Leporinus* cf. *friderici sensu stricto* clade using K2p model. Values as percentage.
