# Supplementary figures and images for: Molecular Evidences of a Hidden Complex Scenario in Leporinus cf. friderici
Source: Front Genet. 2018 Feb 15;9:47. doi: 10.3389/fgene.2018.00047 (PMC5818402; doi:10.3389/fgene.2018.00047)

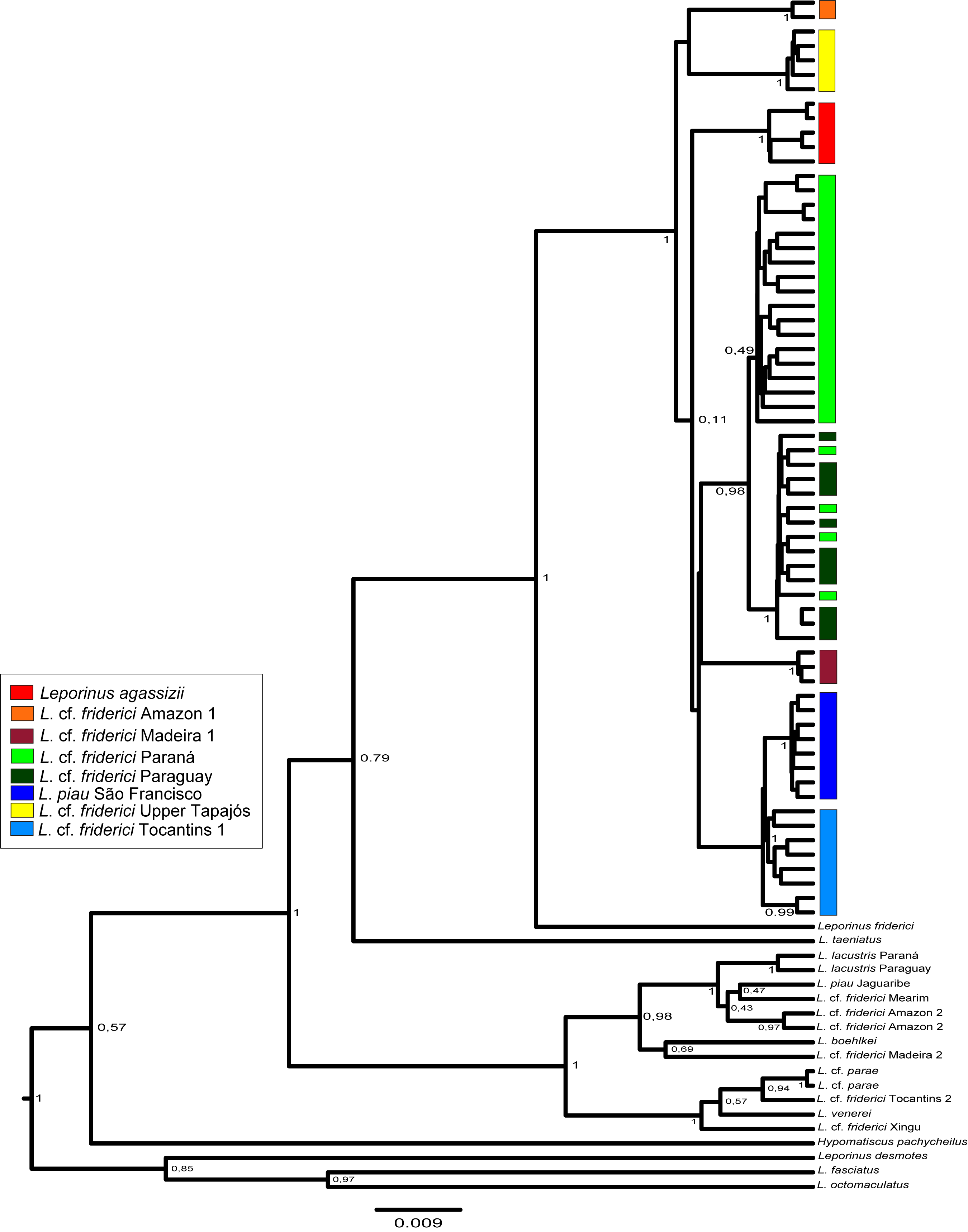

Supplement: FIGURE S1 — Bayesian tree for the cytochrome oxidase subunit 1 (COI) gene. Values on nodes represent the posterior probability. [file Image_1.TIF]

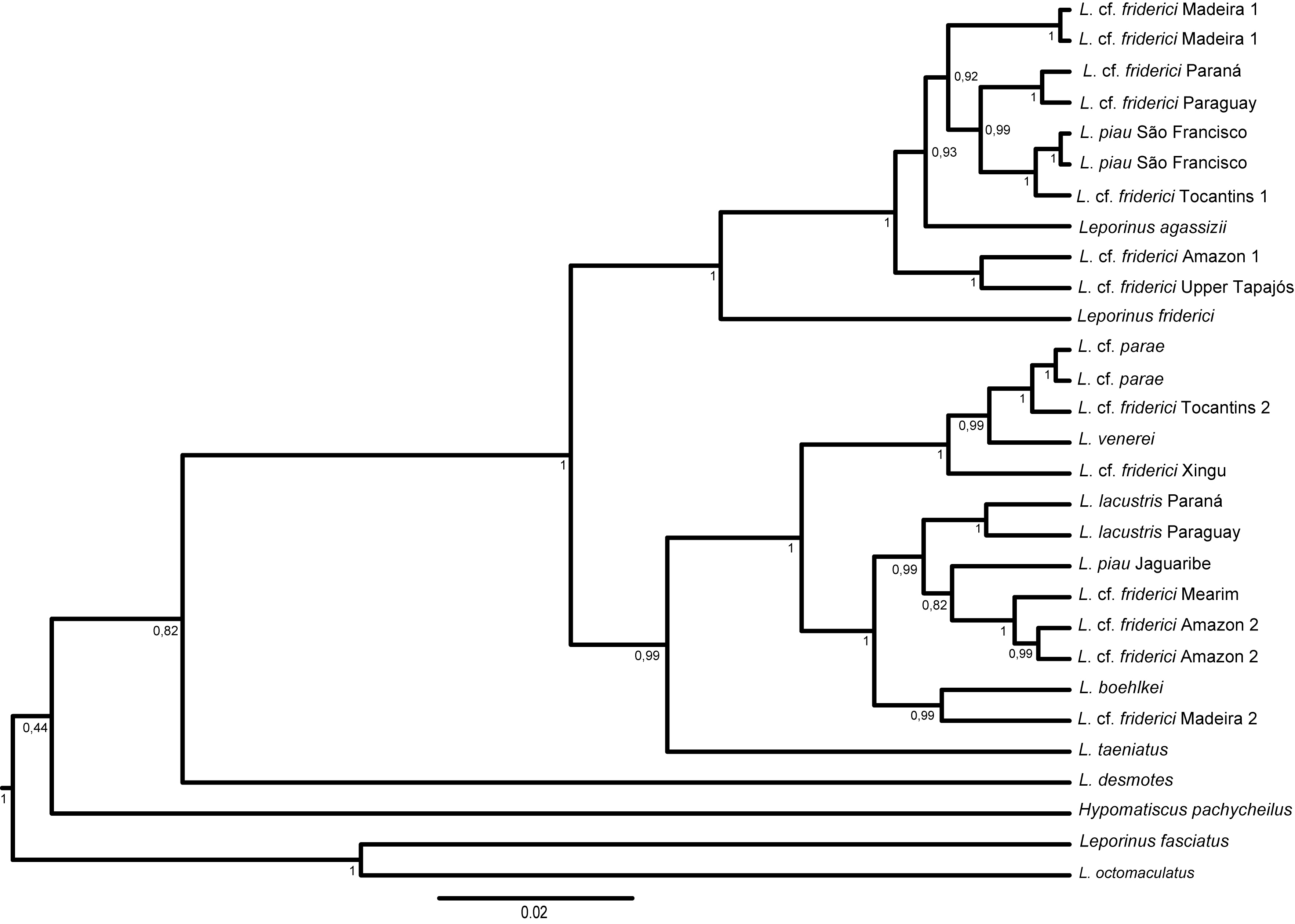

Supplement: FIGURE S2 — Bayesian tree for the cytochrome b (Cytb) gene. Values on nodes represent the posterior probability. [file Image_2.TIF]

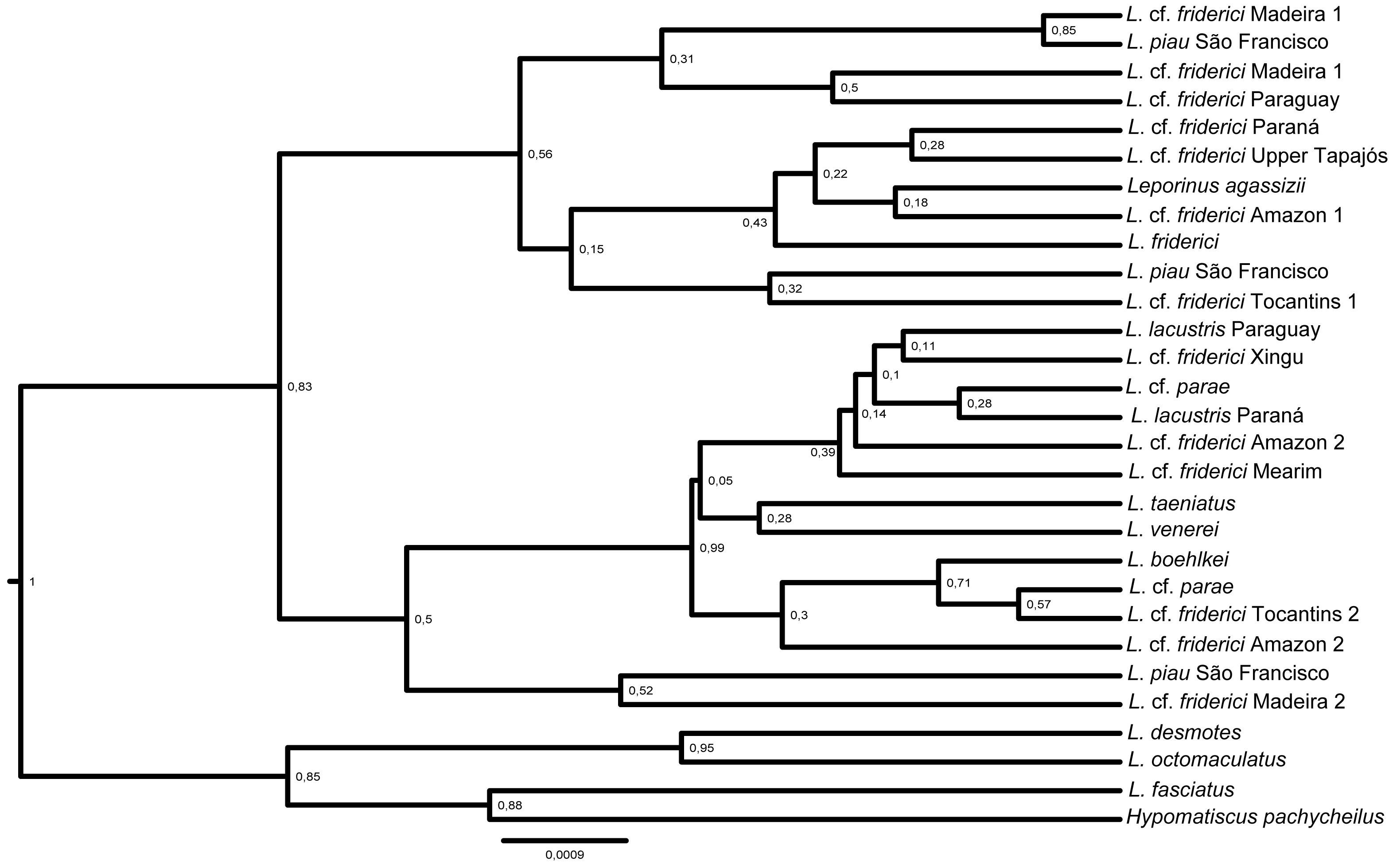

Supplement: FIGURE S3 — Bayesian tree for the myosin heavy chain 6 cardiac muscle alpha (Myh6) gene. Values on nodes represent the posterior probability. [file Image_3.TIF]

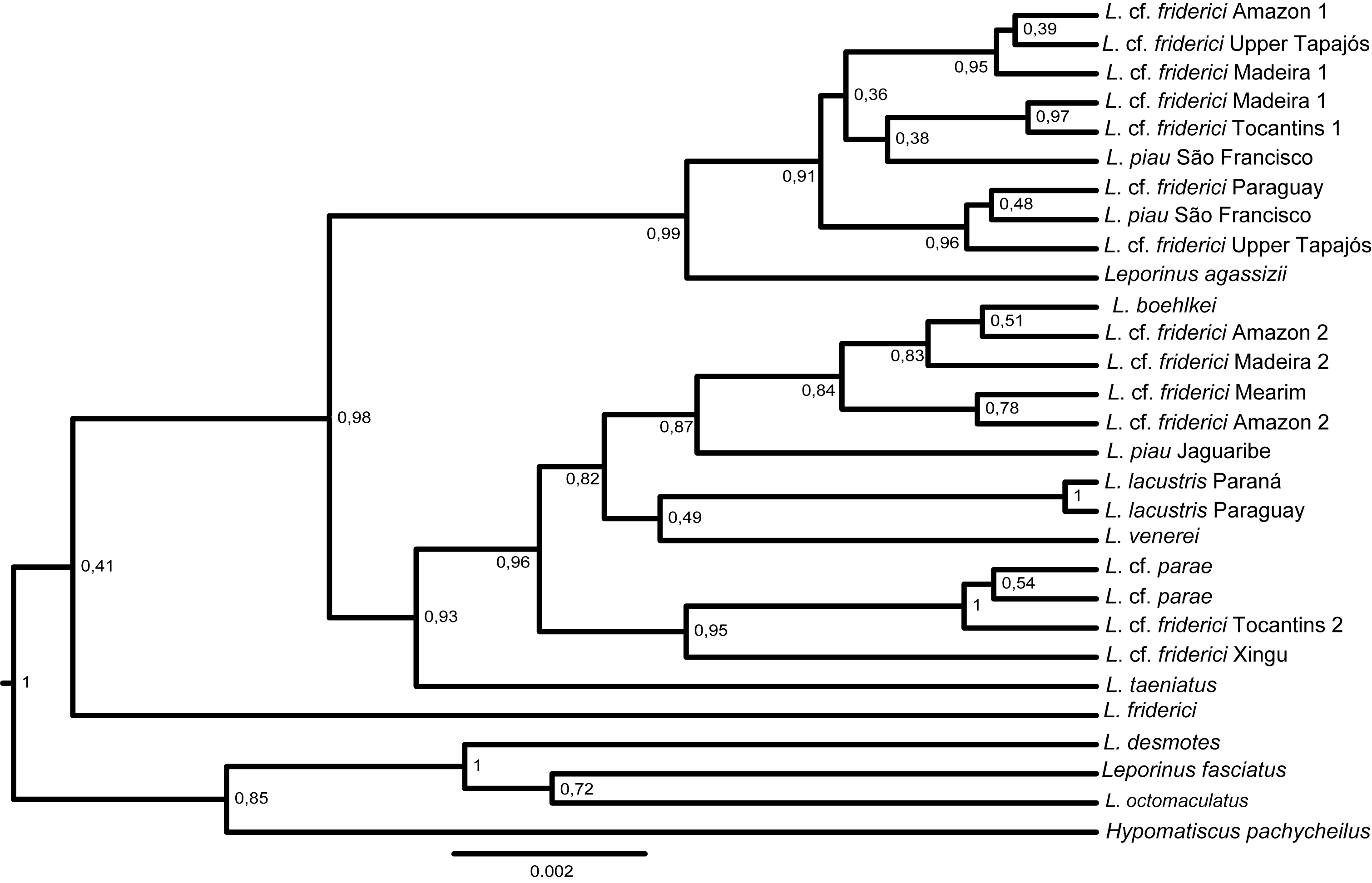

Supplement: FIGURE S4 — Bayesian tree for the recombination activating gene 1 (RAG1) gene. Values on nodes represent the posterior probability. [file Image_4.TIF]

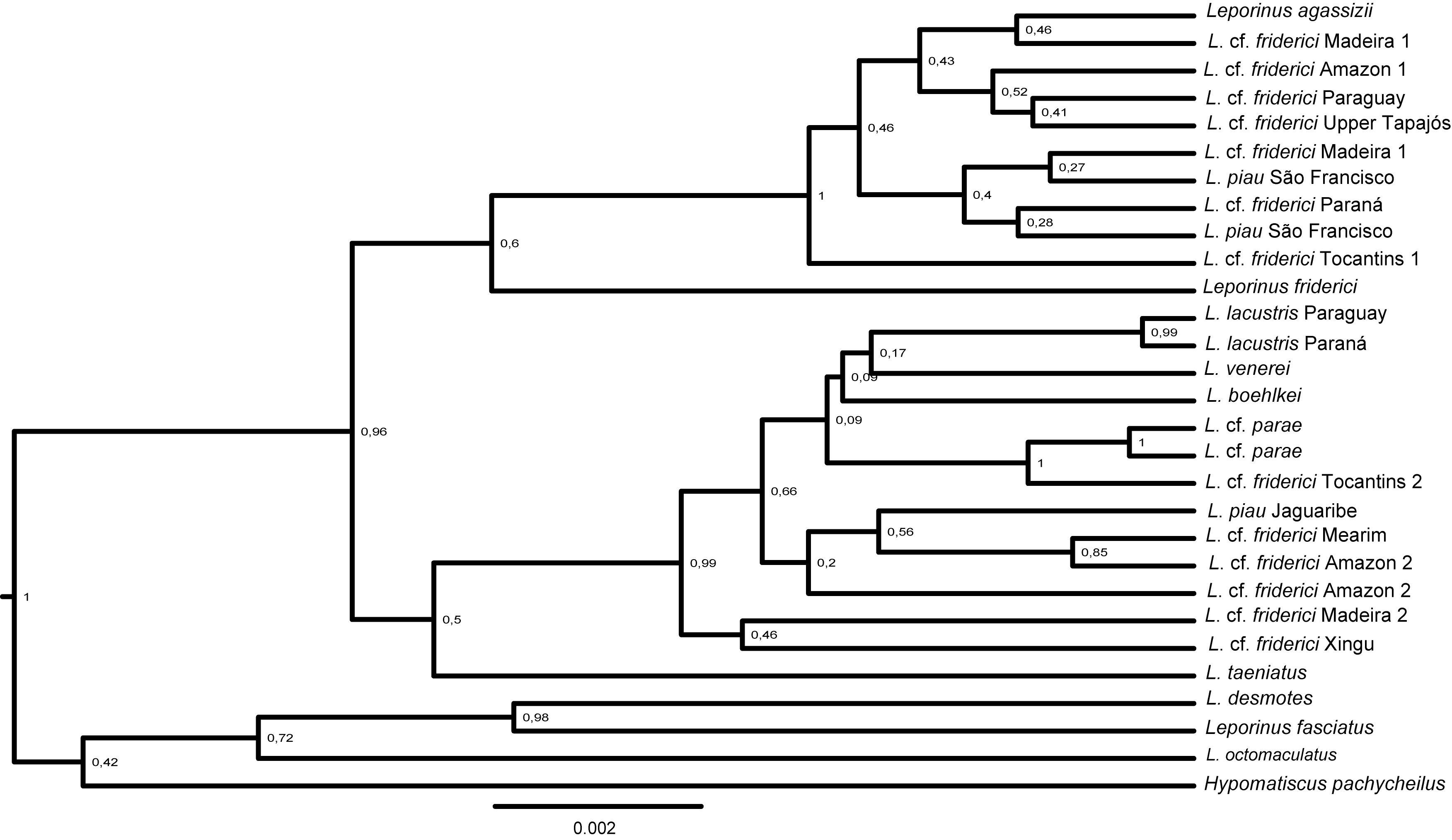

Supplement: FIGURE S5 — Bayesian tree for the recombination activating gene 2 (RAG2) gene. Values on nodes represent the posterior probability. [file Image_5.TIF]
