# Supplementary material for: Molecular Evidences of a Hidden Complex Scenario in Leporinus cf. friderici
Source: Front Genet. 2018 Feb 15;9:47. doi: 10.3389/fgene.2018.00047 (PMC5818402; doi:10.3389/fgene.2018.00047)
Supplement: TABLE S1 — Vouchers and collection sites for all analyzed Leporinus cf. friderici. [file Table_1.docx]

Table S1. Vouchers and collection sites for all analyzed *Leporinus* cf. *friderici*.

|  |  |  |  | |  | |  | |  | |  |  |  |
| --- | --- | --- | --- | --- | --- | --- | --- | --- | --- | --- | --- | --- | --- |
|  |  | | | Collection | | Hydrographic | |  | |  | | Voucher | |
| Samples | Specimens | | | site (river) | | Basin | | Latitude | | Longitude | | number | |
| L226 | *Leporinus* cf. *friderici* Mearim | | | Pindaré ^1^ | | Mearim | | 3°39'33.79"S | | 45°28'2.81"W | | CPUFMA 2921 | |
| L743 | *Leporinus* cf. *friderici* Amazon 1 | | | Jamanxim ^2^ | | Tapajós | | 7º3'52.0''S | | 55º26'28.0''W | | MZUSP 097513 | |
| AECI018-10 | *Leporinus* cf. *friderici* Amazon 1 | | | Amazon ^3^ | | Amazon | | 3º17'13.9''S | | 60º1'87''W | | M3-34 | |
| L258 | *Leporinus* cf. *friderici* Amazon 2 | | | Amazon ^4^ | | Amazon | | 2º13'52.70''S | | 45º19'15.46''W | | - | |
| L515 | *Leporinus* cf. *friderici* Amazon 2 | | | Sampaio lake ^5^ | | Madeira | | 3°50’9.40’’S | | 59°10’43’’W | | UFRO - I 1003 | |
| L742 | *Leporinus* cf*. friderici* Xingu | | | 13 de maio ^6^ | | Xingu | | 8°43’41’’S | | 55°1’38’’W | | MZUSP 097129 | |
| L491 | *Leporinus* cf. *friderici* Madeira 1 | | | Mequéns ^7^ | | Madeira | | 12°51’28.18”S | | 62°10’4.41”W | | - | |
| L516 | *Leporinus* cf. *friderici* Madeira 1 | | | Guaporé ^8^ | | Madeira | | 12°51’16.17”S | | 62°54’5.83”W | | UFRO - 12222 | |
| L073 | *Leporinus* cf. *friderici* Madeira 1 | | | Capivari ^9^ | | Madeira | | 14º42’38.7’’S | | 60º14’56.9’’W | | LISDEBE 6975 | |
| L081 | *Leporinus* cf. *friderici* Madeira 2 | | | Guaporé ^10^ | | Madeira | | 15º12’57.62’’S | | 59º24’14.29’’W | | MZUSP 115358 | |
| L142 | *Leporinus* cf. *friderici* Tocantins 1 | | | Araguaia ^11^ | | Tocantins | | 14º 45’36’’S | | 51º 52’26’’W | | GEPEMA 5477 | |
| L143 | *Leporinus* cf. *friderici* Tocantins 1 | | | Araguaia ^11^ | | Tocantins | | 14º 45’36’’S | | 51º 52’26’’W | | - | |
| L144 | *Leporinus* cf. *friderici* Tocantins 1 | | | Araguaia ^11^ | | Tocantins | | 14º 45’36’’S | | 51º 52’26’’W | | - | |
| L388 | *Leporinus* cf. *friderici* Tocantins 1 | | | Araguaia ^12^ | | Tocantins | | 15º53’42.37”S | | 52º15’16.46”W | | GEPEMA 6115 | |
| L390 | *Leporinus* cf. *friderici* Tocantins 1 | | | Araguaia ^12^ | | Tocantins | | 15º53’42.37”S | | 52º15’16.46”W | | GEPEMA 6117 | |
| L391 | *Leporinus* cf. *friderici* Tocantins 1 | | | Araguaia ^12^ | | Tocantins | | 15º53’42.37”S | | 52º15’16.46”W | | GEPEMA 6118 | |
| L392 | *Leporinus* cf. *friderici* Tocantins 1 | | | Araguaia ^12^ | | Tocantins | | 15º53’42.37”S | | 52º15’16.46”W | | GEPEMA 6119 | |
| L393 | *Leporinus* cf. *friderici* Tocantins 1 | | | Araguaia ^12^ | | Tocantins | | 15º53’42.37”S | | 52º15’16.46”W | | GEPEMA 6120 | |
| L173 | *Leporinus* cf. *friderici* Tocantins 2 | | | Araguaia ^13^ | | Tocantins | | 15º53’42.37”S | | 52°9’44.40”W | | MZUSP 115359 | |
| L030 | *Leporinus* cf. *friderici* Paraguay | | | Bandeirantes ^14^ | | Paraguay | | 14º48’55.3’’S | | 57º06’20.8’’W | | - | |
| L019 | *Leporinus* cf. *friderici* Paraguay | | | Bandeirantes ^14^ | | Paraguay | | 14º48’55.3’’S | | 57º06’20.8’’W | | - | |
| L027 | *Leporinus* cf. *friderici* Paraguay | | | Bandeirantes ^14^ | | Paraguay | | 14º48’55.3’’S | | 57º06’20.8’’W | | - | |
| L029 | *Leporinus* cf. *friderici* Paraguay | | | Bandeirantes ^14^ | | Paraguay | | 14º48’55.3’’S | | 57º06’20.8’’W | | - | |
| L034 | *Leporinus* cf. *friderici* Paraguay | | | Sepotuba ^15^ | | Paraguay | | 14º36’32’8’’S | | 57º44’04.6’’W | | - | |
| L037 | *Leporinus* cf. *friderici* Paraguay | | | Sepotuba ^15^ | | Paraguay | | 14º36’32’8’’S | | 57º44’04.6’’W | | - | |
| L040 | *Leporinus* cf. *friderici* Paraguay | | | Sepotuba ^15^ | | Paraguay | | 14º36’32’8’’S | | 57º44’04.6’’W | | - | |
| L044 | *Leporinus* cf. *friderici* Paraguay | | | Sepotuba ^15^ | | Paraguay | | 14º36’32’8’’S | | 57º44’04.6’’W | | - | |
| L047 | *Leporinus* cf. *friderici* Paraguay | | | Sepotuba ^15^ | | Paraguay | | 14º36’32’8’’S | | 57º44’04.6’’W | | - | |
| L049 | *Leporinus* cf. *friderici* Paraguay | | | Sepotuba ^15^ | | Paraguay | | 14º36’32’8’’S | | 57º44’04.6’’W | | - | |
| L041 | *Leporinus* cf. *friderici* Paraguay | | | Sepotuba ^15^ | | Paraguay | | 14º36’32’8’’S | | 57º44’04.6’’W | | MZUSP – 113985 | |
| L1049 | *Leporinus* cf. *friderici* Upper Tapajós | | | Arinos ^16^ | | Tapajós | | 11°16’51.42”S | | 57°33’39.38”W | | - | |
| L1050 | *Leporinus* cf. *friderici* Upper Tapajós | | | Sangue ^17^ | | Tapajós | | 11°42’22.75”S | | 58°3’00.11”W | | - | |
| L1051 | *Leporinus* cf. *friderici* Upper Tapajós | | | Vermelho ^18^ | | Tapajós | | 10°45’38.9”S | | 58°21’34.6”W | | - | |
| L1052 | *Leporinus* cf. *friderici* Upper Tapajós | | | Juruena ^19^ | | Tapajós | | 9°52’45.4”S | | 58°14’06”W | | - | |
| L739 | *Leporinus* cf. *friderici* Upper Tapajós | | | Renato ^20^ | | Tapajós | | 11º4’24.0’’S | | 55º14’35.0’’W | | MZUSP 099126 | |
| L018 | *Leporinus* cf. *friderici* Paraná | | | Turvo ^21^ | | Paraná | | 20º25’24.0’’S | | 49º15’16.6’’W | | MZUSP 113983 | |
| L271 | *Leporinus* cf. *friderici* Paraná | | | Paraná ^22^ | | Paraná | | 23º21’38.81”S | | 53º45’12.30”W | | - | |
| L272 | *Leporinus* cf. *friderici* Paraná | | | Paraná ^22^ | | Paraná | | 23º21’38.81”S | | 53º45’12.30”W | | - | |
| L273 | *Leporinus* cf. *friderici* Paraná | | | Paraná ^22^ | | Paraná | | 23º21’38.81”S | | 53º45’12.30”W | | - | |
| L274 | *Leporinus* cf. *friderici* Paraná | | | Paraná ^22^ | | Paraná | | 23º21’38.81”S | | 53º45’12.30”W | | - | |
| L275 | *Leporinus* cf. *friderici* Paraná | | | Paraná ^22^ | | Paraná | | 23º21’38.81”S | | 53º45’12.30”W | | - | |
| L277 | *Leporinus* cf. *friderici* Paraná | | | Paraná ^22^ | | Paraná | | 23º21’38.81”S | | 53º45’12.30”W | | - | |
| L278 | *Leporinus* cf. *friderici* Paraná | | | Paraná ^22^ | | Paraná | | 23º21’38.81”S | | 53º45’12.30”W | | - | |
| L279 | *Leporinus* cf. *friderici* Paraná | | | Paraná ^22^ | | Paraná | | 23º21’38.81”S | | 53º45’12.30”W | | - | |
| L280 | *Leporinus* cf. *friderici* Paraná | | | Paraná ^22^ | | Paraná | | 23º21’38.81”S | | 53º45’12.30”W | | - | |
| L281 | *Leporinus* cf. *friderici* Paraná | | | Paraná ^22^ | | Paraná | | 23º21’38.81”S | | 53º45’12.30”W | | - | |
| L367 | *Leporinus* cf. *friderici* Paraná | | | Paraná ^23^ | | Paraná | | 21º08’30.37” S | | 51º48’43.80”W | | - | |
| L374 | *Leporinus* cf. *friderici* Paraná | | | Paraná ^23^ | | Paraná | | 21º08’30.37” S | | 51º48’43.80”W | | - | |
| L400 | *Leporinus* cf. *friderici* Paraná | | | Paraná ^24^ | | Paraná | | 23º25’12”S | | 53º54’23”W | | - | |
| L403 | *Leporinus* cf. *friderici* Paraná | | | Paraná ^24^ | | Paraná | | 23º25’12”S | | 53º54’23”W | | - | |
| L405 | *Leporinus* cf. *friderici* Paraná | | | Paraná ^24^ | | Paraná | | 23º25’12”S | | 53º54’23”W | | - | |
| L428 | *Leporinus* cf. *friderici* Paraná | | | Paraná ^24^ | | Paraná | | 23º25’12”S | | 53º54’23”W | | - | |
| L730 | *Leporinus* cf. *friderici* Paraná | | | Jacaré-Pepira ^25^ | | Paraná | | 22º14’14.12’’S | | 48º15’49.38’’W | | - | |
| L823 | *Leporinus* cf. *friderici* Paraná | | | Piracicaba ^26^ | | Paraná | | 22º43’10.29’’S | | 47º39’19’’W | | - | |
| L886 | *Leporinus* cf. *friderici* Paraná | | | Mogi-Guaçu ^27^ | | Paraná | | 21º55’38.6”S | | 47º21’58.4”W | | - | |
| L887 | *Leporinus* cf. *friderici* Paraná | | | Mogi-Guaçu ^27^ | | Paraná | | 21º55’38.6”S | | 47º21’58.4”W | | - | |
| LBPV44953 | *Leporinus* cf. *friderici* Paraná | | | Invinhema ^28^ | | Paraná | | 21ºS | | 54°W | | - | |
| L678 | *Leporinus agassizii* | | | Nanay ^29^ | | Amazon | | 3º49’55”S | | 73º21’44”W | | MUSM 47362 | |
| L679 | *Leporinus agassizii* | | | Nanay ^29^ | | Amazon | | 3º49’55”S | | 73º21’44”W | | MUSM 47362 | |
| L680 | *Leporinus agassizii* | | | Nanay ^29^ | | Amazon | | 3º49’55”S | | 73º21’44”W | | MUSM 47362 | |
| L681 | *Leporinus agassizii* | | | Nanay ^29^ | | Amazon | | 3º49’55”S | | 73º21’44”W | | MUSM 47362 | |
| L682 | *Leporinus agassizii* | | | Nanay ^29^ | | Amazon | | 3º49’55”S | | 73º21’44”W | | MUSM 47362 | |
| L322 | *Leporinus piau* São Francisco | | | Urucuia ^30^ | | São Francisco | | 16º09’57.87”S | | 45º40’21.75”W | | - | |
| L323 | *Leporinus piau* São Francisco | | | Urucuia ^30^ | | São Francisco | | 16º09’57.87”S | | 45º40’21.75”W | | - | |
| L330 | *Leporinus piau* São Francisco | | | Urucuia ^30^ | | São Francisco | | 16º09’57.87”S | | 45º40’21.75”W | | MCP44093 | |
| L331 | *Leporinus piau* São Francisco | | | Pandeiros ^31^ | | São Francisco | | 15º 40’18’’S | | 44º38’12.3’’W | | MCP44837 | |
| L332 | *Leporinus piau* São Francisco | | | Lagoa da Presa ^32^ | | São Francisco | | 19º44’36”S | | 45º27’23”W | | MCP44763 | |
| L333 | *Leporinus piau* São Francisco | | | Lagoa da Presa ^32^ | | São Francisco | | 19º44’36”S | | 45º27’23”W | | MCP44763 | |
| L334 | *Leporinus piau* São Francisco | | | Lagoa da Presa ^32^ | | São Francisco | | 19º44’36”S | | 45º27’23”W | | MCP44763 | |
| L335 | *Leporinus piau* São Francisco | | | Lagoa da Presa ^32^ | | São Francisco | | 19º44’36”S | | 45º27’23”W | | MCP44763 | |
| CPUFMA, Coleção de Peixes, Universidade Federal do Maranhão  LISDEBE, Laboratório de Ictiologia Sistemática, Departamento de Ecologia e Biologia Evolutiva, Universidade Federal de São Carlos  GEPEMA, Grupo de Estudos em Peixes do Médio Araguaia, Campus Universitário do Médio Araguaia, Universidade Federal do Mato Grosso  M3-34, University of Oviedo  MCP, Museu de Ciências e Tecnologia, Pontifícia Universidade Católica de Minas Gerais  MZUSP, Museu de Zoologia, Universidade de São Paulo | | | | | | | | | | | | |  |
| MUSM, Museo de Historia Natural, Universidad Nacional Mayor de San Marcos, Lima  UFRO, Universidade Federal de Rondônia | | | | | | | | | | | | |  |
