# Supplementary material for: Molecular Evidences of a Hidden Complex Scenario in Leporinus cf. friderici
Source: Front Genet. 2018 Feb 15;9:47. doi: 10.3389/fgene.2018.00047 (PMC5818402; doi:10.3389/fgene.2018.00047)
Supplement: TABLE S2 — GenBank accession numbers for all analyzed species. [file Table_2.docx]

Table S2. GenBank accession numbers for all analyzed species.

|  |  |  | |  |  |  |  |
| --- | --- | --- | --- | --- | --- | --- | --- |
|  |  |  | |  | GenBank acession numbers |  |  |
| Sample | Species | COI | | CytB | Myh6 | RAG1 | RAG2 |
| L226 | *Leporinus* cf. *friderici* Mearim | MF664226 | | MF664289 | MF664304 | MF664319 | MF664334 |
| L743 | *Leporinus* cf. *friderici* Amazon 1 | MF664227 | | MF664290 | MF664305 | MF664320 | MF664335 |
| AECI018-10 | *Leporinus* cf. *friderici* Amazon 1 | AECI018-10 ¹ | | - | - | - | - |
| L258 | *Leporinus* cf. *friderici* Amazon 2 | MF664228 | | MF664291 | MF664306 | MF664321 | MF664336 |
| L742 | *Leporinus* cf. *friderici* Xingu | MF664229 | | MF664292 | MF664307 | MF664322 | MF664337 |
| L491 | *Leporinus* cf. *friderici* Madeira 1 | MF664230 | | MF664293 | MF664308 | MF664323 | MF664338 |
| L516 | *Leporinus* cf. *friderici* Madeira 1 | MF664231 | | MF664294 | MF664309 | MF664324 | MF664339 |
| L073 | *Leporinus* cf. *friderici* Madeira 1 | MF664232 | | - | - | - | - |
| L081 | *Leporinus cf. friderici* Madeira 2 | MF664233 | | MF664295 | MF664310 | MF664325 | MF664340 |
| L515 | *Leporinus* cf. *friderici* Madeira 2 | MF664234 | | MF664296 | MF664311 | MF664326 | MF664341 |
| L142 | *Leporinus* cf. *friderici* Tocantins 1 | MF664235 | | MF664297 | MF664312 | MF664327 | MF664342 |
| L143 | *Leporinus* cf. *friderici* Tocantins 1 | MF664236 | | - | - | - | - |
| L144 | *Leporinus* cf. *friderici* Tocantins 1 | MF664237 | | - | - | - | - |
| L388 | *Leporinus* cf. *friderici* Tocantins 1 | MF664238 | | - | - | - | - |
| L390 | *Leporinus* cf. *friderici* Tocantins 1 | MF664239 | | - | - | - | - |
| L391 | *Leporinus* cf. *friderici* Tocantins 1 | MF664240 | | - | - | - | - |
| L392 | *Leporinus* cf. *friderici* Tocantins 1 | MF664241 | | - | - | - | - |
| L393 | *Leporinus* cf. *friderici* Tocantins 1 | MF664242 | | - | - | - | - |
| L173 | *Leporinus* cf. *friderici* Tocantins 2 | MF664243 | | MF664298 | MF664313 | MF664328 | MF664343 |
| L030 | *Leporinus* cf. *friderici* Paraguay | MF664244 | | - | - | - | - |
| L034 | *Leporinus* cf. *friderici* Paraguay | MF664245 | | - | - | - | - |
| L037 | *Leporinus* cf. *friderici* Paraguay | MF664246 | | - | - | - | - |
| L040 | *Leporinus* cf. *friderici* Paraguay | MF664247 | | - | - | - | - |
| L044 | *Leporinus* cf. *friderici* Paraguay | MF664248 | | - | - | - | - |
| L047 | *Leporinus* cf. *friderici* Paraguay | MF664249 | | - | - | - | - |
| L049 | *Leporinus* cf. *friderici* Paraguay | MF664250 | | - | - | - | - |
| L041 | *Leporinus* cf. *friderici* Paraguay | KF568983² | | KF569026 ² | KF569155 ² | KF569069 ² | KF569112 ² |
| L019 | *Leporinus* cf. *friderici* Paraguay | MF664251 | | - | - | - | - |
| L027 | *Leporinus* cf. *friderici* Paraguay | MF664252 | | - | - | - | - |
| L029 | *Leporinus* cf. *friderici* Paraguay | MF664253 | | - | - | - | - |
| L1049 | *Leporinus* cf. *friderici* Upper Tapajós | MF664254 | | - | - | - | - |
| L1050 | *Leporinus* cf. *friderici* Upper Tapajós | MF664255 | | - | - | - | - |
| L1051 | *Leporinus* cf. *friderici* Upper Tapajós | MF664256 | | - | - | - | - |
| L1052 | *Leporinus* cf. *friderici* Upper Tapajós | MF664257 | | - | - | - | - |
| L739 | *Leporinus* cf. *friderici* Upper Tapajós | MF664258 | | MF664299 | MF664314 | MF664329 | MF664344 |
| L018 | *Leporinus* cf. *friderici* Paraná | KF568982² | | KF569025 ² | KF569154 ² | KF569154 ² | KF569154 ² |
| LBPV-44953 | *Leporinus* cf. *friderici* Paraná | JN988988² | | - | - | - | - |
| L271 | *Leporinus* cf. *friderici* Paraná | MF664259 | | - | - | - | - |
| L272 | *Leporinus* cf. *friderici* Paraná | MF664260 | | - | - | - | - |
| L273 | *Leporinus* cf. *friderici* Paraná | MF664261 | | - | - | - | - |
| L274 | *Leporinus* cf. *friderici* Paraná | MF664262 | | - | - | - | - |
| L275 | *Leporinus* cf. *friderici* Paraná | MF664263 | | - | - | - | - |
| L277 | *Leporinus* cf. *friderici* Paraná | MF664264 | | - | - | - | - |
| L278 | *Leporinus* cf. *friderici* Paraná | MF664265 | | - | - | - | - |
| L279 | *Leporinus* cf. *friderici* Paraná | MF664266 | | - | - | - | - |
| L280 | *Leporinus* cf. *friderici* Paraná | MF664267 | | - | - | - | - |
| L281 | *Leporinus* cf. *friderici* Paraná | MF664268 | | - | - | - | - |
| L367 | *Leporinus* cf. *friderici* Paraná | MF664269 | | - | - | - | - |
| L374 | *Leporinus* cf. *friderici* Paraná | MF664270 | | - | - | - | - |
| L400 | *Leporinus* cf. *friderici* Paraná | MF664271 | | - | - | - | - |
| L403 | *Leporinus* cf. *friderici* Paraná | MF664272 | | - | - | - | - |
| L405 | *Leporinus* cf. *friderici* Paraná | MF664273 | | - | - | - | - |
| L428 | *Leporinus* cf. *friderici* Paraná | MF664274 | | - | - | - | - |
| L730 | *Leporinus* cf. *friderici* Paraná | MF664275 | | - | - | - | - |
| L823 | *Leporinus* cf. *friderici* Paraná | MF664276 | | - | - | - | - |
| L886 | *Leporinus* cf. *friderici* Paraná | MF664277 | | - | - | - | - |
| L887 | *Leporinus* cf. *friderici* Paraná | MF664278 | | - | - | - | - |
| L678 | *Leporinus agassizii* | MF664279 | | - | - | - | - |
| L679 | *Leporinus agassizii* | MF664280 | | MF664300 | MF664315 | MF664330 | MF664345 |
| L680 | *Leporinus agassizii* | MF664281 | | - | - | - | - |
| L681 | *Leporinus agassizii* | MF664282 | | - | - | - | - |
| L682 | *Leporinus agassizii* | MF664283 | | - | - | - | - |
| L910 | *Leporinus boehlkei* | MF664284 | | MF664301 | MF664316 | MF664331 | MF664346 |
| L376 | *Leporinus piau* Jaguaribe | MF664285 | | MF664302 | MF664317 | MF664332 | MF664347 |
| L322 | *Leporinus piau* São Francisco | KF568990² | | KF569033 ² | KF569162 ² | KF569076 ² | KF569119 ² |
| L323 | *Leporinus piau* São Francisco | MF664286 | | MF664303 | MF664318 | MF664333 | MF664348 |
| L330 | *Leporinus piau* São Francisco | HM405143 ² | | - | - | - | - |
| L331 | *Leporinus piau* São Francisco | HM405144 ² | | - | - | - | - |
| L332 | *Leporinus piau* São Francisco | HM405145 ² | | - | - | - | - |
| L333 | *Leporinus piau* São Francisco | HM405146 ² | | - | - | - | - |
| L334 | *Leporinus piau* São Francisco | MF664287 | | - | - | - | - |
| L335 | *Leporinus piau* São Francisco | MF664288 | | - | - | - | - |
| L141 | *Leporinus desmotes* | KF568979 ² | | KF569022 ² | KF569151 ² | KF569065 ² | KF569108 ² |
| L496 | *Leporinus* *fasciatus* | KF568981 ² | | KF569024 ² | KF569153 ² | KF569067 ² | KF569110 ² |
| L088 | *Leporinus lacustris* Paraguay | KF568985 ² | | KF569028 ² | KF569157 ² | KF569071 ² | KF569114 ² |
| L213 | *Leporinus lacustris* Paraná | KF568986 ² | | KF569029 ² | KF569158 ² | KF569072 ² | KF569115 ² |
| L021 | *Leporinus octomaculatus* | KF568988 ² | | KF569031 ² | KF569160 ² | KF569074 ² | KF569117 ² |
| L527 | *Leporinus* cf. *parae* | KF568999 ² | | KF569042 ² | KF569171 ² | KF569085 ² | KF569128 ² |
| L631 | *Leporinus* cf. *parae* | MF677847 | | MF677848 | MF677849 | MF677850 | MF677851 |
| L356 | *Leporinus taeniatus* | KF568996 ² | | KF569039 ² | KF569168 ² | KF569082 ² | KF569125 ² |
| L185 | *Leporinus venerei* | KF569001 ² | | KF569044 ² | KF569173 ² | KF569087 ² | KF569130 ² |
| L479 | *Hypomasticus pachycheilus* | KF568973 ² | | KF569016 ² | KF569145 ² | KF569059 ² | KF569102 ² |
| ANSP 189264 | *Leporinus friderici* | KX086747 ² | | KX086812 ² | KX086872 ² | KX086958 ² | KX086985 ² |
|  |  |  | |  |  |  |  |
| ¹ obtained from BOLDSYTEMS | | |  |  |  |  |  |
| ² Obtained from GenBank | | |  |  |  |  |  |
| COI, C*ytochrome Oxidase Subunit 1* | | |  |  |  |  |  |
| Cytb, C*ytochrome b* | | |  |  |  |  |  |
| Myh6, M*yosin Heavy Chain 6 Cardiac Muscle Alpha* | | |  |  |  |  |  |
| RAG1, R*ecombination Activating Gene 1* | | |  |  |  |  |  |
| RAG2, R*ecombination Activating Gene 2* | | |  |  |  |  |  |
