# Supplementary material for: Molecular Evidences of a Hidden Complex Scenario in Leporinus cf. friderici
Source: Front Genet. 2018 Feb 15;9:47. doi: 10.3389/fgene.2018.00047 (PMC5818402; doi:10.3389/fgene.2018.00047)
Supplement: TABLE S3 — Pairwise mean genetic distances values inter and intra-MOTU (in bold) Leporinus cf. friderici sensu stricto clade using K2p model. Values as percentage. [file Table_3.docx]

Table S3. Pairwise mean genetic distances values inter and intra-MOTU (in bold) *Leporinus* cf. *friderici sensu* *stricto* clade using K2p model. Values as percentage.

|  |  |  |  |  |  |  |  |  |
| --- | --- | --- | --- | --- | --- | --- | --- | --- |
|  | *L agassizii* | *L.* cf. *friderici* | *L.* cf. *friderici* | *L.* cf. *friderici* | *L.* cf. *friderici* | *L.* *piau* | *L.* cf. *friderici* | *L.* cf. *friderici* |
|  |  | Amazon 1 | Madeira 1 | Paraná | Paraguay | São Francisco | Upper Tapajós | Tocantins 1 |
| *L.agassizii* | **0.5** |  |  |  |  |  |  |  |
| *L.* cf. *friderici*Amazon 1 | 1.5 | **0.2** |  |  |  |  |  |  |
| *L.* cf. *friderici* Madeira 1 | 1.6 | 1.0 | **0.0** |  |  |  |  |  |
| *L.* cf. *friderici* Paraná | 1.5 | 0.9 | 1.1 | **0.2** |  |  |  |  |
| *L.* cf. *friderici* Paraguay | 1.8 | 1.3 | 1.4 | 0.5 | **0.2** |  |  |  |
| *L.* *piau* São Francisco | 1.9 | 1.4 | 1.1 | 1.1 | 1.4 | **0.0** |  |  |
| *L.* cf. *friderici* Upper Tapajós | 2.4 | 1.7 | 2.1 | 2.0 | 2.4 | 2.1 | **0.1** |  |
| *L.* cf. *friderici* Tocantins 1 | 2.0 | 1.4 | 1.1 | 1.1 | 1.4 | 0.4 | 2.2 | **0.4** |
| *Leporinus friderici* (Suriname) | 5.0 | 4.9 | 5.0 | 4.8 | 4.5 | 5.4 | 5.2 | 5.3 |
|  |  |  |  |  |  |  |  |  |
